# DAILY VARIABILITY OF MULTI-DIMENSIONAL SUBJECTIVE AGE ACROSS THE ADULT LIFE-COURSE: IMPACT OF SCORING METHODS

**DOI:** 10.1093/geroni/igae098.4150

**Published:** 2024-12-31

**Authors:** Shally Zhou, Brooke Brady, Lidan Zheng, Kaarin Anstey

**Affiliations:** University of New South Wales, Sydney, New South Wales, Australia; University of New South Wales, Sydney, New South Wales, Australia; University of New South Wales, Sydney, New South Wales, Australia; University of New South Wales, Sydney, New South Wales, Australia

## Abstract

Subjective age (SA), measured as how old you feel, is associated with positive health and developmental outcomes. Despite the increased use of multi-dimensional items, there is great heterogeneity in how it is implemented. SA measures often use different scoring methods which may impact subsequent analyses and interpretations. To understand the daily fluctuations of multi-dimensional SA and evaluate the impact of scoring differences, 210 participants aged 18 to 84 (M = 54.50, SD = 18.03, 60.95% female) reported six SAs over two week-long bursts of daily surveys. Common SA scoring methods including raw, discrepancy, proportional and composite average scores were compared. Multi-level models demonstrate chronological age is a significant predictor for all scoring methods across all SA dimensions. Examining raw and discrepancy scores, younger participants felt older than their chronological age, but this difference decreased and reversed with increasing age leading to older participants feeling younger. Proportional scores reveal the percentage difference between felt SA and chronological age reduced 0.17% every year, indicating felt discrepancies were largest at both extremes of the sample. Furthermore, intercepts and significant associations varied across scoring methods and SA dimensions for sex, expectations regarding aging, self-perceptions of aging, daily stress, and daily positive and negative affect. Exploring multi-dimensional SA provides deeper insights into life-course variability, and a more nuanced understanding of ageing. Scoring approaches led to somewhat different interpretations in a life-course sample. Future studies are encouraged to consider the study’s design and main research question when determining the most appropriate questions and scoring method.

